# Synthetic Approaches and Pharmacological Activity of 1,3,4-Oxadiazoles: A Review of the Literature from 2000–2012

**DOI:** 10.3390/molecules170910192

**Published:** 2012-08-27

**Authors:** Cledualdo Soares de Oliveira, Bruno Freitas Lira, José Maria Barbosa-Filho, Jorge Gonçalo Fernandez Lorenzo, Petrônio Filgueiras de Athayde-Filho

**Affiliations:** 1 Department of Chemistry, Federal University of Paraíba, 58051-900 João Pessoa-PB, Brazil; Email: aldoscarchi@yahoo.com.br (C.S.O.); brunofrlira@hotmail.com (B.F.L.); 2 Laboratory of Pharmaceutical Technology, Federal University of Paraíba, 58051-900 João Pessoa-PB, Brazil; Email: jbarbosa@ltf.ufpb.br (J.M.B.-F.); jgflorenzo@hotmail.com (J.G.F.L.)

**Keywords:** 1,3,4-oxadiazole, synthesis methods, pharmacological activity, review

## Abstract

This review provides readers with an overview of the main synthetic methodologies for 1,3,4-oxadiazole derivatives, and of their broad spectrum of pharmacological activities as reported over the past twelve years.

## 1. Introduction

**Figure 1:**
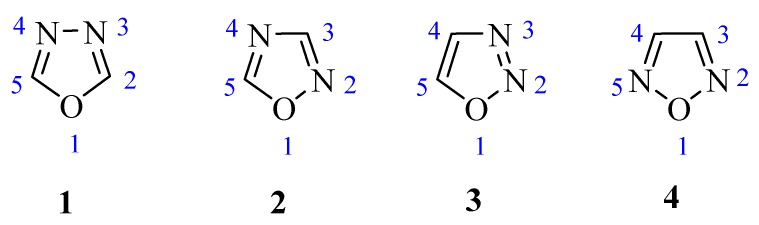
Isomers of oxadiazole.

1,3,4-Oxadiazole (**1**, Figure 1) is a heterocyclic compound containing an oxygen atom and two nitrogen atoms in a five-membered ring. It is derived from furan by substitution of two methylene groups (=CH) with two pyridine type nitrogens (-N=) [1,2]. There are three known isomers: 1,2,4-oxadiazole (**2**), 1,2,3-oxadiazole (**3**) and 1,2,5-oxadiazole (**4**) (Figure 1). However, 1,3,4-oxadiazole and 1,2,4-oxadiazole are better known, and more widely studied by researchers because of their many important chemical and biological properties.

**Figure 2:**
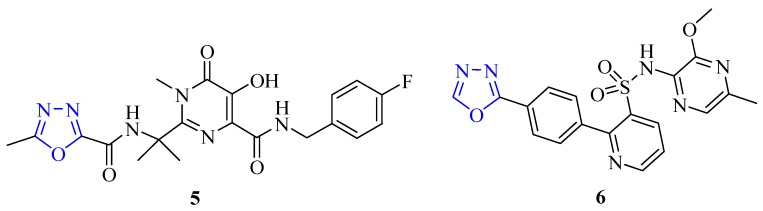
Structures of raltegravir and zibotentan, drugs that are in late stage clinical development.

Among heterocyclic compounds, 1,3,4-oxadiazole has become an important construction motif for the development of new drugs. Compounds containing 1,3,4-oxadiazole cores have a broad biological activity spectrum including antibacterial, antifungal, analgesic, anti-inflammatory, antiviral, anticancer, antihypertensive, anticonvulsant, and anti-diabetic properties. They have also attracted interest in medicinal chemistry as surrogates (bioisosteres) for carboxylic acids, esters and carboxamides [2]. The ability of 1,3,4-oxadiazole heterocyclic compounds to undergo various chemical reactions has made them important for molecule planning because of their privileged structure, which has enormous biological potential. Two examples of compounds containing the 1,3,4-oxadiazole unit currently used in clinical medicine are: Raltegravir^®^ (**5**), an antiretroviral drug [3] and Zibotentan^®^ (**6**) an anticancer agent [4] (Figure 2).

**Figure 3:**
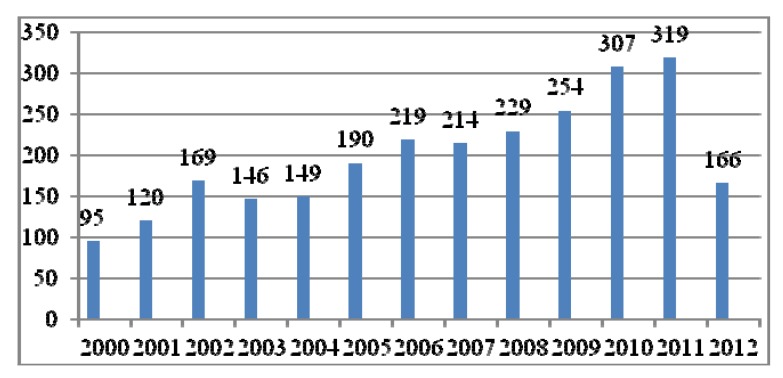
Number of publications in the last twelve years involving 1,3,4-oxadiazole.

The synthesis of novel 1,3,4-oxadiazole derivatives, and investigation of their chemical properties and biological behavior has accelerated in the last two decades. In recent years the number of scientific studies with these compounds has increased considerably. Considering the period from 2002 to 2012, the Scifinder Scholar database records 2,577 references to 1,3,4-oxadiazole, demonstrating its relevance for heterocyclic chemistry. Figure 3 shows the number of publications over the past twelve years involving 1,3,4-oxadiazole. The graph is of course not linear, there is a decrease from 2002 (169 articles) to 2003 (146 articles), and then a gradual increase from 2003 to 2006 (219 articles), again a small decline from 2006 to 2007 (214 articles), and an increase from 2007 to 2011 (319 articles) [5].

Taking into account the importance of these compounds to both heterocyclic and medicinal chemistry, we have decided to present the main synthesis approaches used for obtaining the heterocyclic nucleus, as well as the broad spectrum of pharmacological activities reported in the literature over the past twelve years.

## 2. Methods of Synthesis for 2,5-Disubstituted-1,3,4-oxadiazoles

### 2.1. Methods of Synthesis for 5-Substituted-2-amino-1,3,4-oxadiazoles

**Scheme 1:**
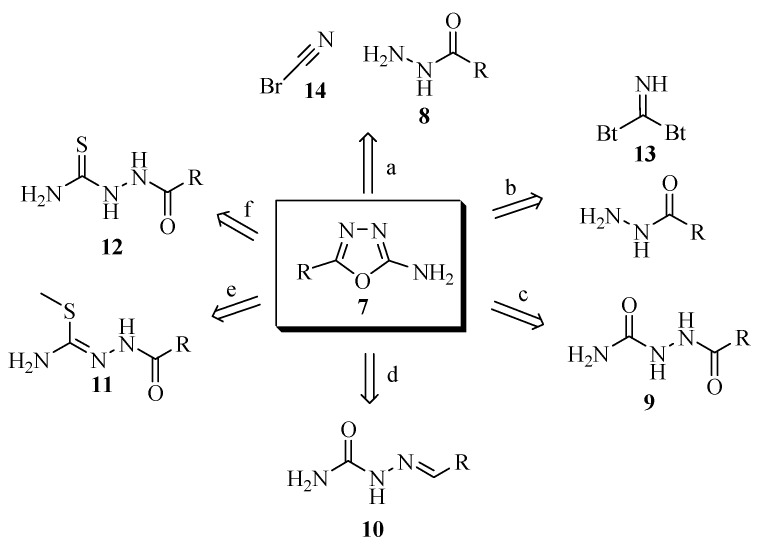
Retrosynthetic analysis of 5-substituted-2-amino-1,3,4-oxadiazole.

A few of the methods reported in the literature for the preparation of 5-substituted-2-amino-1,3,4-oxadiazoles **7** are outlined in Scheme 1. Methods **a** and **b** use the acylhydrazide intermediate **8**, readily prepared from the corresponding ester and hydrazine hydrate, which can react with cyanogen bromide (**14**), or di(benzotriazol-1-yl)methanimine (**23**). Dehydration of acylsemicarbazide **9** has also been used extensively, although more stringent conditions are necessary (method **c**). Acylthiosemicarbazide intermediates **11** and **12** have been used in different routes to obtain the desired heterocycles through oxidative cyclization reactions with iodine at elevated temperatures, or by carbodiimide derivatives (methods **e** and **f**). Semicarbazones **10**, being versatile intermediates, can easily be cyclized to the corresponding 1,3,4-oxadiazoles (method **d**).

**Scheme 2:**
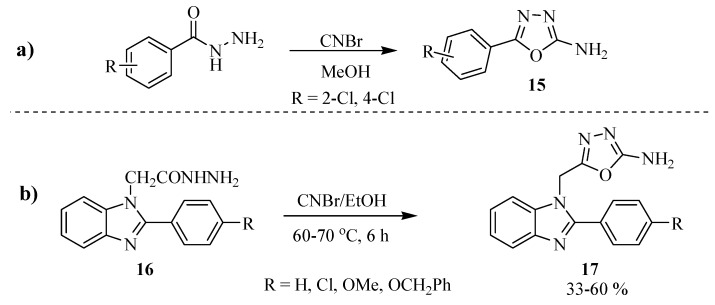
5-Aryl-2-amino-1,3,4-oxadiazole obtained from acylhydrazides and cyanogen bromide.

Using approach (**a**) of Scheme 1, Patel and Patel [6] synthesized 5-aryl-2-amino-1,3,4-oxadiazole compounds **15** in yields of 62 to 70%. These compounds were used as intermediates for the synthesis of new quinazolinone derivatives (Entry **a**, Scheme 2). Kerimov and co-workers [7] synthesized a new series of 2-amino-1,3,4-oxadiazoles **17** carrying a benzimidazole moiety in 33%–60% yield from the reaction between 2-(2-(4-substituted-phenyl)-1*H*-benzo[d]imidazol-1-yl)acetohydrazide (**16**) and cyanogen bromide, (Entry **b**, Scheme 2).

**Scheme 3:**
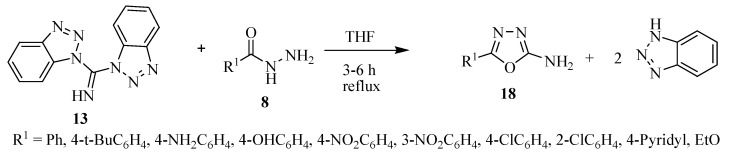
5-Aryl-2-amino-1,3,4-oxadiazole obtained from acylhydrazides and di(benzotriazol-1-yl)methanimine.

Katritzky and co-workers [8] have prepared 5-aryl-2-amino-1,3,4-oxadiazole compounds **18** (Scheme 3) in excellent yields from the reaction between di(benzotriazol-1-yl)methanimine (**13**) and arylhydrazides **8** using approach (**b**) of Scheme 1.

**Scheme 4:**
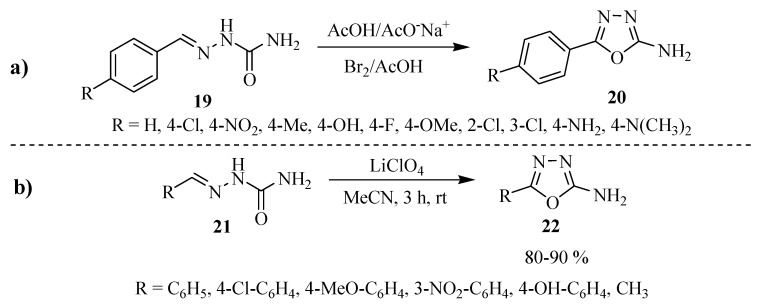
5-Substituted-1,3,4-oxadiazol-2-amines from of cyclization reaction of semicarbazones.

The oxidative cyclization of semicarbazones **19** (Scheme 4) with bromine in acetic acid is one of the approaches frequently used for the preparation of 5-substituted-1,3,4-oxadiazol-2-amines **20** (Entry **a**, Scheme 4) [9,10]. Electro-cyclization of semicarbazones **21** to their corresponding 5-aryl-2-amino-1,3,4-oxadiazoles **22** has emerged as an alternative method (Entry **b**, Scheme 4) [11,12].

**Scheme 5:**
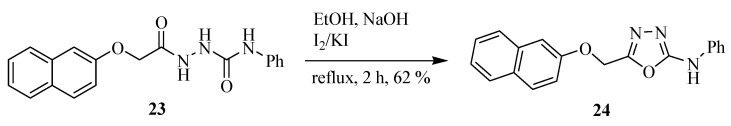
Synthesis of 1,3,4-oxadiazol-2-amines from of cyclization reaction of acylthiosemicarbazides with iodine.

Another interesting approach to the synthesis of 5-substituted-2-amino-1,3,4-oxadiazoles is the cyclization reaction of acylthiosemicarbazides using iodine as the oxidizing agent. El-Sayed and co-workers [13] reported the synthesis of 5-((naphthalen-2-yloxy)methyl)-*N*-phenyl-1,3,4-oxadiazol-2-amine (**24**) in 62% yield, by heating compound **23** in ethanol in the presence of sodium hydroxide and iodine in potassium iodide (Scheme 5).

**Scheme 6:**
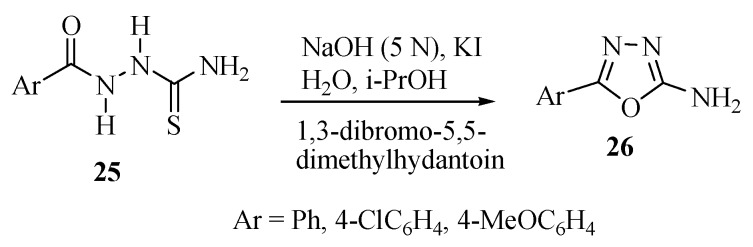
Synthesis of 5-aryl-2-amino-1,3,4-oxadiazole from acylthiosemicarbazide and 1,3-dibromo-5,5-dimethylhydantoin.

Rivera and co-workers [14] reported that 1,3-dibromo-5,5-dimethylhydantoin is an effective oxidizing agent for cyclization reactions of acylthiosemicarbazide. Compounds **25** were cyclized to 5-aryl-2-amino-1,3,4-oxadiazoles **26** in excellent yield (Scheme 6). The main advantage of this method is that the reagents used are commercially cheap and safe to work with. Further, it is applicable to large scale synthesis where other oxidizing agents cannot be used.

**Scheme 7:**
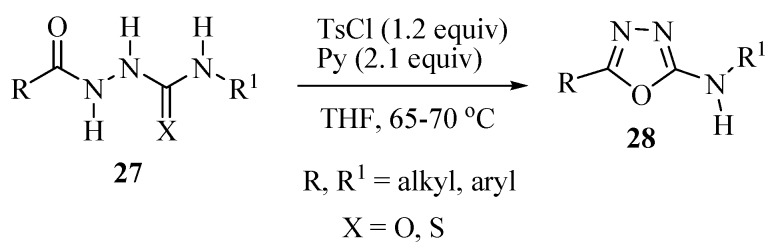
Synthesis of 5-aryl-2-amino-1,3,4-oxadiazole from acylthiosemicarbazide and tosyl chloride.

In general the 5-aryl(alkyl)-2-amino-1,3,4-oxadiazoles can be prepared by dehydration of derivatives of semicarbazides or thiosemicarbazides using POCl_3_ as dehydrating agent. Alternative reagents that activate the carbonyl group have also been used. Accordingly, Dolman and co-workers [15] reported a new method of synthesis for 5-aryl(alkyl)-2-amino-1,3,4-oxadiazoles **28** from acylsemicarbazides **27** (X=O) and acylthiosemicarbazides **27** (X=S) mediated by tosyl chloride. Yields of 97%–99% were obtained when using thiosemicarbazide derivatives which are more reactive than the corresponding semicarbazide derivatives (Scheme 7).

### 2.2. Methods of Synthesis for 5-Substituted-1,3,4-oxadiazole-2-thiols

**Scheme 8:**
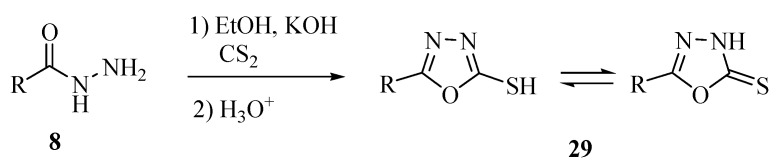
Synthesis of 5-substituted-1,3,4-oxadiazole-2-thiols.

The main synthesis route for 5-substituted-1,3,4-oxadiazole-2-thiol(thione)s **29** involves an initial reaction between an acylhydrazide **8** and carbon disulfide in an basic alcohol solution, followed by acidification of the reaction mixture (Scheme 8). A large number of 1,3,4-oxadiazole derivatives prepared by this route have been reported in recent years. The existence of thiol-thione tautomerism is known for the compounds **29**, and one of the forms usually predominates [16].

**Figure 4:**
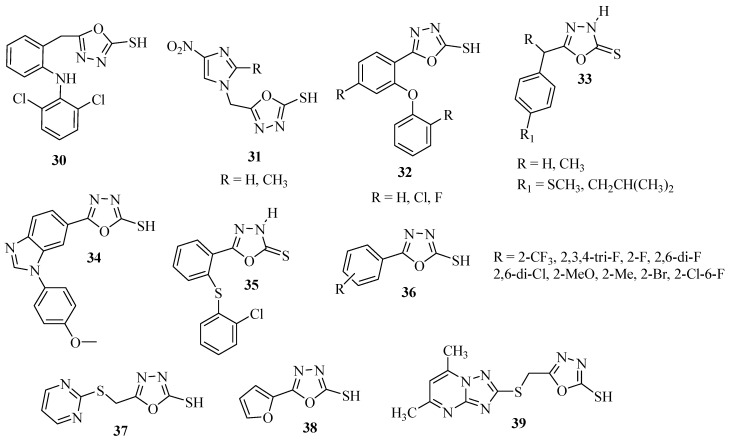
5-Aryl-1,3,4-oxadiazole-2-thiols obtained by reaction of acylhydrazide with carbon disulfide.

The compounds (**30**) [17], (**31**) [18], (**32**) [19], (**33**) [20], (**34**) [21], (**35**) [22], (**36**) [23], (**37**) [24], (**38**) [16] and (**39**) [25] (Figure 4) are just some of many compounds of this class prepared using the synthetic route shown in Scheme 8.

### 2.3. Methods of Synthesis for 2,5-Diaryl(alkyl)-1,3,4-oxadiazole

**Scheme 9:**
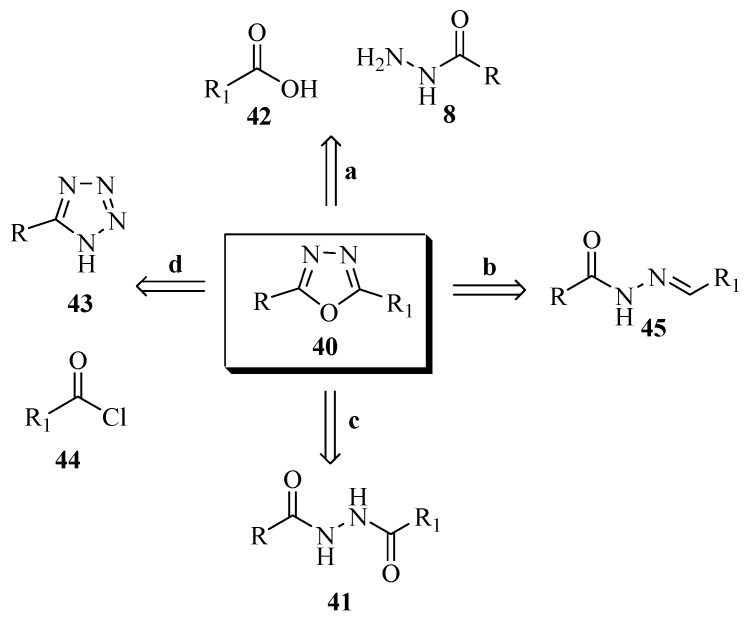
Retrosynthetic analysis of 2,5-diaryl(alkyl)-1,3,4-oxadiazoles.

Several methods of synthesis have been reported in the literature for the preparation of both symmetrical and asymmetrical 2,5-diaryl(alkyl)-1,3,4-oxadiazoles **40** (Scheme 9). One of the most popular methods involves the cyclodehydration of 1,2-diacylhydrazines **41** using phosphorous oxychloride (POCl_3_) as a dehydrating agent, path (**c**) of Scheme 9. Other dehydrating agents commonly used are sulfuric acid, phosphoric acid, trifluoroacetic acid, phosphorus pentachloride, phosphorus pentoxide, thionyl chloride, and milder reagents such as carbodiimide derivatives, TsCl/pyridine, trimethylsilyl chloride, Ph_3_O/Tf_2_O, PPh_3_/CX_4_ (X = Cl, Br, I), and Burgess reagent. Other important routes for obtaining either symmetrical (R = R_1_) or asymmetric (R ≠ R_1_), 2,5-diaryl(alkyl)-1,3,4-oxadiazoles **40** are reactions of acylhydrazides **8** with aromatic carboxylic acids **42** (**a**), oxidative cyclization of acylhydrazones **45** (**b**), and the reaction of tetrazoles **43** with acid chlorides **44** in the presence of pyridine, path (**d**) (Scheme 9).

**Scheme 10:**
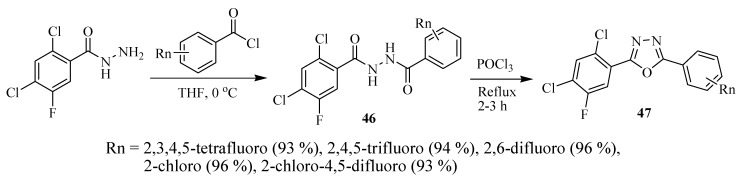
Synthesis of asymmetrical 2-(2,4-dichloro-5-fluorophenyl)-5-aryl-1,3,4-oxadiazoles.

**Scheme 11:**
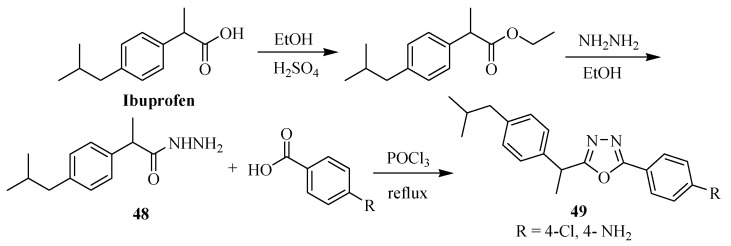
Synthesis of 2,5-dissubstituted-1,3,4-oxadiazole derivatives of ibuprofen.

The asymmetrical 5-(2,4-dichloro-5-flurophenyl)-2-(aryl)-1,3,4-oxadiazole compounds **47** were prepared in two steps (Scheme 10) by refluxing the corresponding diacylhydrazines **46** with phosphorus oxychloride as reported by Zheng and co-workers [26]. If not intending to isolate the intermediate diacylhydrazine, generally the one-pot reaction of a carboxylic acid with acylhydrazide and POCl_3_ as dehydrating agent is used (see approach **a**, Scheme 9). Similarly, Amir and Kumar [27] reported the synthesis of novel 2,5-disubstituted-1,3,4-oxadiazole derivatives **49** beginning with the anti-inflammatory drug ibuprofen as a starting material (Scheme 11). Phosphorus oxychloride (POCl_3_) was used as a dehydrating agent in the reaction of acylhydrazide **48** with substituted aromatic carboxylic acids. 

**Scheme 12:**
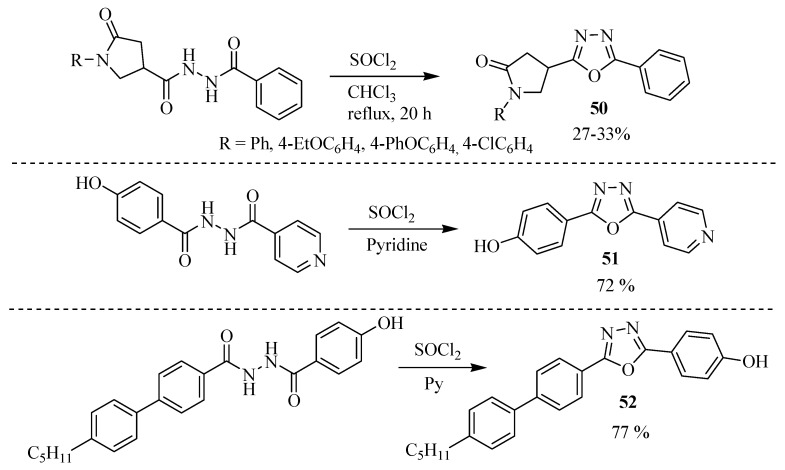
Cyclization of diacylhydrazine with thionyl chloride. **50** [28], **51** [29] and **52** [30].

Another dehydrating agent normally used for the dehydration of diacylhydrazines is thionyl chloride; Scheme 12 outlines some examples. 

**Scheme 13:**
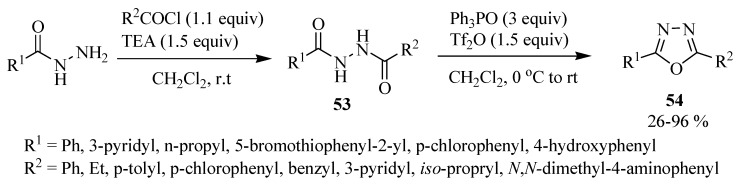
Cyclodehydration of diacylhydrazine using triphenylphosphine oxide and triflic anhydride.

The use of POCl_3_ requires care because it is very toxic and corrosive. Less dangerous reagents (that are easier to work with) than POCl_3_ have arisen in recent years. For example, Bostrom and co-workers [2] synthesized 2,5-disubstituted-1,3,4-oxadiazole compounds **54** by cyclodehydration of diacylhydrazine **53** using triphenylphosphine oxide (3 equivalents), and triflic anhydride (1.5 equivalents), obtaining from 26 to 96% yields (Scheme 13).

**Scheme 14:**
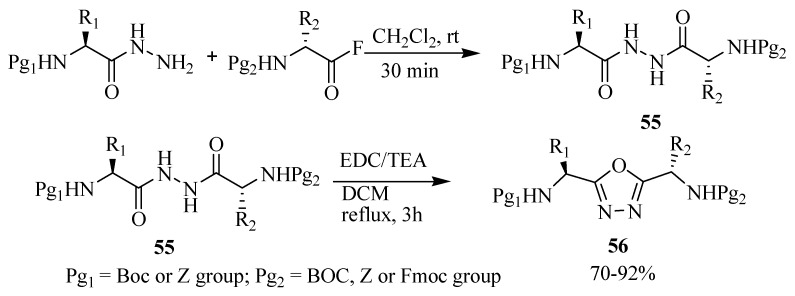
Cyclodehydration reaction of diacylhydrazines using EDC.

Nagendra and co-workers [31] reported the synthesis of novel orthogonally protected 1,3,4-oxadiazole **56**, tethered dipeptide mimetics, by cyclodehydration of diacylhydrazine **55** using 1-ethyl-3-(3-dimethylaminopropyl) carbodiimide (EDC) as a dehydration agent, obtaining a 70%–92% yields (Scheme 14).

**Scheme 15:**
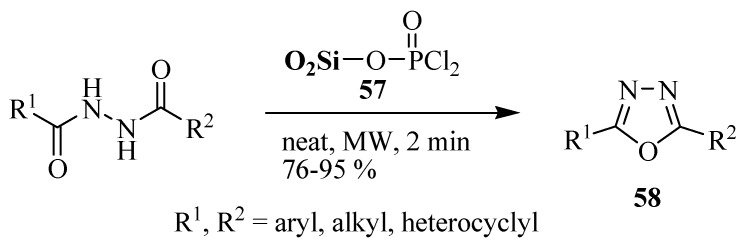
Synthesis of 2,5-disubstituted 1,3,4-oxadiazoles from 1,2-diacylhydrazines and silica-supported dichlorophosphate.

Li and co-workers [32] reported that silica-supported dichlorophosphate is an efficient cyclodehydration agent for the synthesis of 2,5-disubstituted 1,3,4-oxadiazoles **58** from 1,2-diacylhydrazines in solvent-free medium under microwave irradiation. This protocol was suitable for the synthesis of alkyl, aryl, and heterocyclic substituted symmetrical and unsymmetrical 1,3,4-oxadiazoles, and has the specific advantages of no corrosion or environmental pollution, an accelerated rate, high yield and a simple work-up procedure (Scheme 15).

**Scheme 16:**
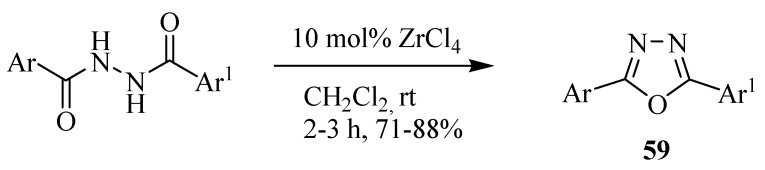
Synthesis of 2,5-disubstituted 1,3,4-oxadiazoles from 1,2-diacylhydrazines and zirconium(IV) chloride.

Sharma and co-workers [33] developed a simple generalized method for the synthesis of 1,3,4-oxadiazoles **59** from diacylhydrazines using inexpensive ZrCl_4_ as a catalyst. Advantages over the existing methods include higher yields, shorter reaction times, and a simple experimental procedure (Scheme 16).

**Scheme 17:**
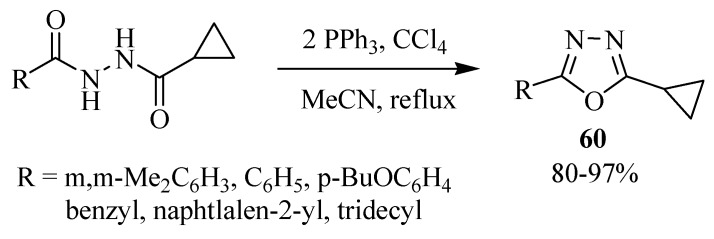
Effect of halogens in the formation of 1,3,4-oxadiazoles.

Yang and Shi [34] reported the effect of halogens in a Robinson-Gabriel type reaction of cyclopropane-carboxylic acid *N'*-substituted-hydrazides with PPh_3_/CX_4_ (X = Cl, Br, I) as dehydration agents resulting in the formation of 1,3,4-oxadiazoles **60** (Scheme 17).

**Scheme 18:**
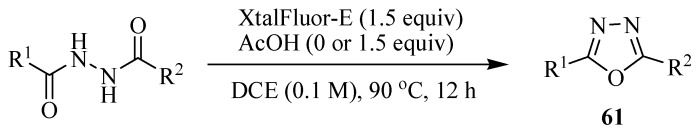
Preparation of 1,3,4-oxadiazoles from 1,2-diacylhydrazines using XtalFluor-E.

Pouliot and co-workers [35] reported the use of diethylaminodifluorosulfinium tetrafluoroborate ([Et_2_NSF_2_]BF_4_), XtalFluor-E, as a new cyclodehydration agent for the preparation of 1,3,4-oxadiazoles **61** from 1,2-diacylhydrazines (Scheme 18).

**Scheme 19:**
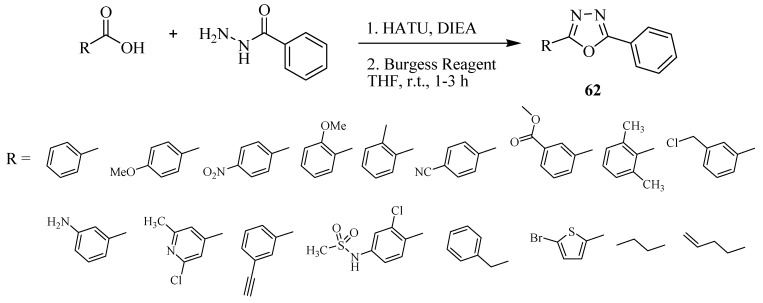
Synthesis of 1,3,4-oxadiazoles from carboxylic acids and hydrazides using HATU and Burgess reagent.

Li and Dickson [36] developed a convenient one-pot protocol for the synthesis of 1,3,4-oxadiazoles **62** from carboxylic acids and hydrazides using HATU as coupling agent and Burgess reagent as dehydrating agent (Scheme 19).

**Scheme 20:**
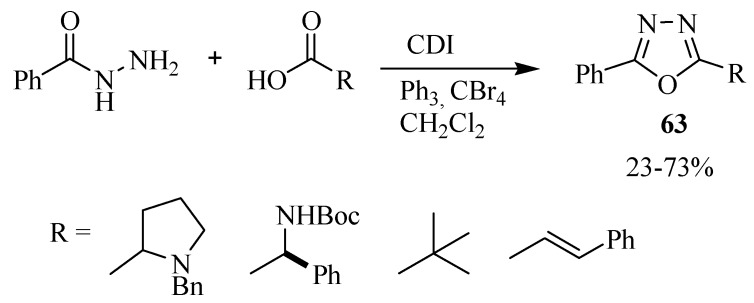
Synthesis of 2,5-dissubstituted-1,3,4-oxadiazoles using CDI and triphenylphosphyne.

Another method for one pot synthesis of 2,5-disubstituted-1,3,4-oxadiazoles **63** from benzohydrazide and carboxylic acid was reported by Rajapakse [37] using the coupling agent 1,1'-carbonyldiimidazole (CDI) and triphenylphosphyne as dehydrating agent (Scheme 20).

**Scheme 21:**
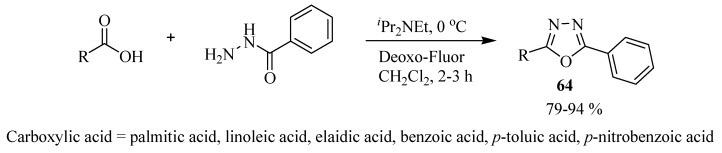
Synthesis of 1,3,4-oxadiazoles using Deoxo-Fluor.

In 2006, Kangani and co-workers [38] described a one-pot direct synthesis of 1,3,4-oxadiazoles **64** in excellent yields from carboxylic acids (1 equiv) and benzohydrazide (2.2 equiv) using Deoxo-Fluor reagent (Scheme 21).

**Scheme 22:**
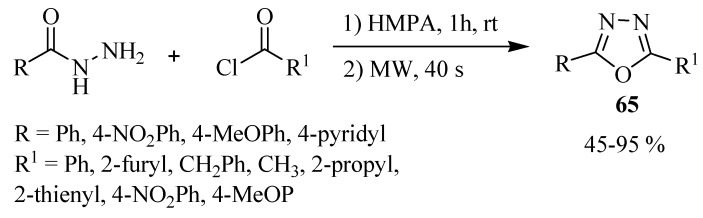
Synthesis of 2,5-disubstituted-1,3,4-oxadiazoles using microwave heating.

A convenient, one pot procedure was also reported for the synthesis of a variety of 2,5-disubstituted-1,3,4-oxadiazoles **65** by condensing monoarylhydrazides with acid chlorides in HMPA solvent under microwave heating. The yields were good to excellent, the process was rapid, and needed no additional acid catalyst or dehydrating reagent (Scheme 22) [39].

**Scheme 23:**
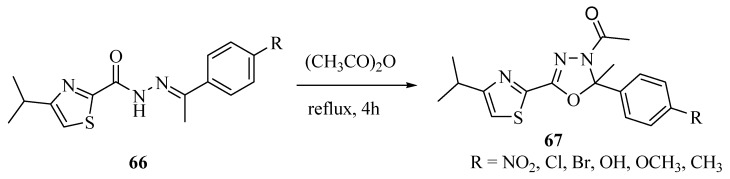
Synthesis of 1,3,4-oxadiazolines from *N*-acylhydrazones using acetic anhydride.

In approach **b** (see Scheme 10), *N*-acylhydrazones generally suffer oxidative cyclization under the action of oxidizing agents such as Br_2_, HgO, KMnO_4_ and acetic anhydride. Other milder oxidizing agents have emerged in recent years such as ammonium cerium nitrate, Cu(OTf)_2_, chloramine-T, trichloroisocyanuric acid and the hypervalent iodines. For example, the reaction of *N*-acylhydrazones **66** treated with acetic anhydride under reflux conditions gives compounds **67** in good yields (Scheme 23) [40].

**Scheme 24:**
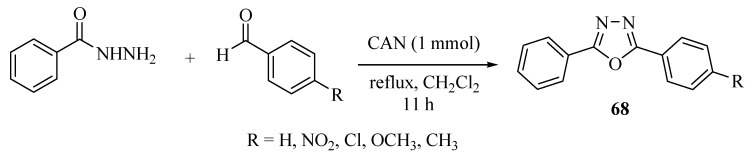
Synthesis of 2,5-diaryl-1,3,4-oxadiazoles from benzohydrazide and aromatic aldehydes.

In 2006, Dabiri and co-workers [41] reported a new procedure for the synthesis of disubstituted oxadiazoles **68** through a one-pot reaction of benzohydrazide, and *para* substituted aromatic aldehydes in the presence of an cerium ammonium nitrate (CAN) and dichloromethane solvent (Scheme 24).

**Scheme 25:**
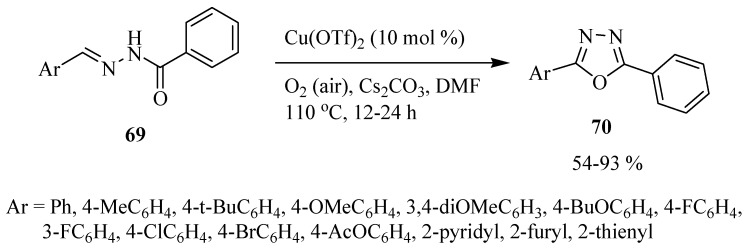
Synthesis of 1,3,4-oxadiazoles from *N*-arylidenearoylhydrazides and Cu(OTf)_2_.

Guin and co-workers [42] reported a direct route to both symmetrical and unsymmetrical 2,5-disubstituted-1,3,4-oxadiazoles **70** by means of an imine C-H functionalization of *N*-arylidenearoylhydrazide **69** using Cu(OTf)_2_ as catalyst (Scheme 25).

**Scheme 26:**
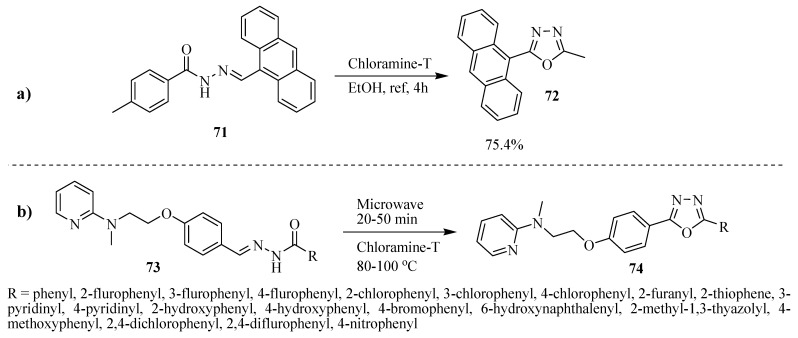
Oxidative cyclization of *N*-acylhydrazones using chloramine-T.

Li and He [43] synthetized the compound 2-(anthracen-9-yl)-5-(p-tolyl)-1,3,4-oxadiazole (**72**) in 75.4% yield from oxidative cyclization of *N*-acylhydrazone **71** using chloramine-T, (Entry **a**, Scheme 26). Gaonkar and co-workers [44] also reported the synthesis of 1,3,4-disubstituted oxadiazoles **74** from the oxidative cyclization of *N*-acylhydrazones **73** with chloramine-T under microwave irradiation, (Entry **b**, Scheme 26).

**Scheme 27:**
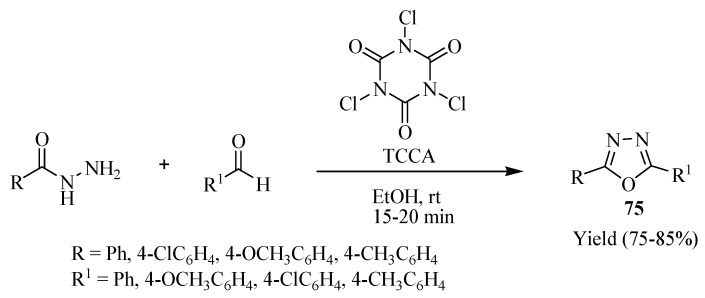
Synthesis of 1,3,4-oxadiazoles using trichloroisocyanuric acid (TCCA).

Pore and co-workers [45] developed an efficient method for one-pot synthesis of unsymmetrical 2,5-disubstituted 1,3,4-oxadiazoles **75** using trichloroisocyanuric acid (TCCA) at ambient temperatures. The main advantages of this method are the mild nature of the synthesis, and the short reaction time (Scheme 27).

**Scheme 28:**
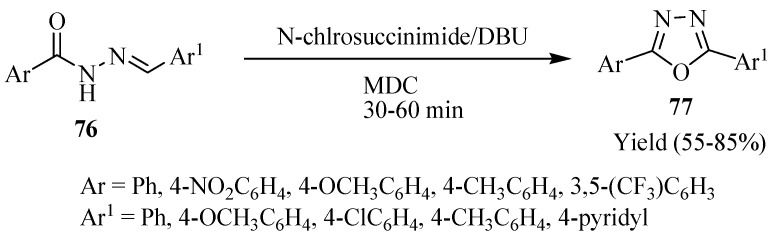
Oxidative cyclization of acylhydrazones using N-chlorosuccinimide and 1,8-diazabicyclo-[5.4.0]undec-7-ene (DBU).

Pardeshi and co-workers [46], using a mixture of *N*-chlorosuccinimide and 1,8-diazabicyclo[5.4.0]undec-7-ene (DBU) oxidatively cyclized structurally diverse acylhydrazones **76**, thereby providing an efficient and convenient method for the synthesis of various 2,5-disubstituted 1,3,4-oxadiazoles **77**. The salient features of this method are the mild reaction conditions, short reaction time, excellent yields, and a simple workup procedure (Scheme 28).

**Scheme 29:**
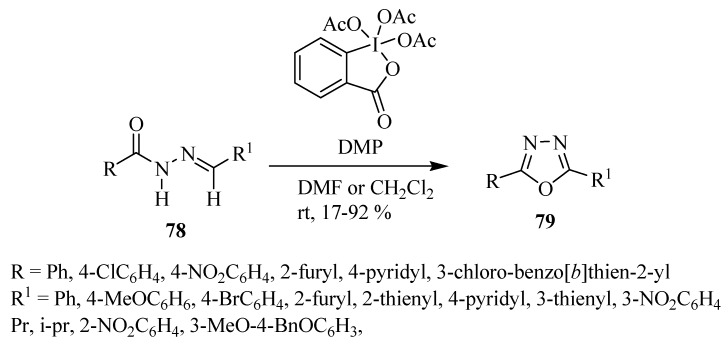
Oxidative cyclization of *N*-acylhydrazones using Dess-Martin periodinane.

Dobrotã and co-workers [47] reported the synthesis of 2,5-disubstituted-1,3,4-oxadiazoles **79**, conveniently prepared by oxidative cyclization of *N*-acylhydrazones **78** through use of an excess of Dess-Martin periodinane under mild conditions (Scheme 29).

**Scheme 30:**
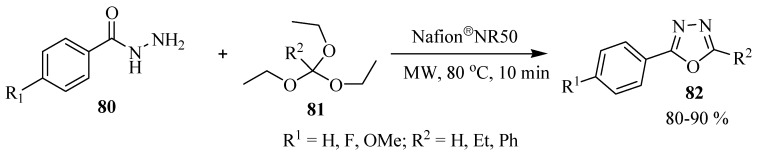
Nafion catalized 1,3,4-oxadiazole synthesis.

Polshettiwar and Varma [48] reported a novel one-pot solvent-free synthesis of 1,3,4-oxadiazoles **82** by condensation of benzohydrazide **80** and triethylorthoalkanates **81** under microwave irradiation, and efficiently catalyzed by Nafion^®^NR50 (solid supported), and phosphorus pentasulfide in alumina (P_4_S_10_/Al_2_O_3_) with excellent yields (Scheme 30).

**Scheme 31:**
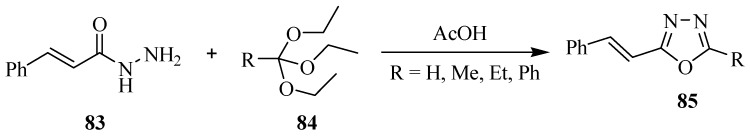
Reaction of cinnamic acid hydrazide with triethyl orthoesters.

Kudelko and Zieliǹski [49] developed an easy and efficient method to synthesize 5-substituted-2-styryl-1,3,4-oxadiazoles **85** from cinnamic acid hydrazide **83** and commercially available triethyl orthoesters **84**. The method provides the desired products rapidly and in high yields making it a useful addition to the existing synthetic procedures (Scheme 31).

**Scheme 32:**
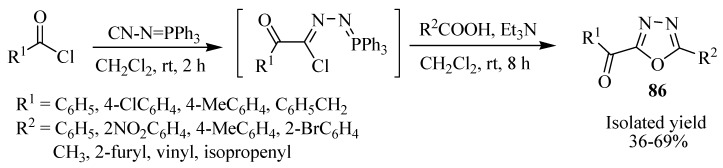
Synthesis α-keto-1,3,4-oxadiazole.

Cui and co-workers [50] reported the synthesis of various α-keto-1,3,4-oxadiazole derivatives through a sequential intermolecular (dehydrochlorination/intramolecular) aza-Wittig reaction of carboxylic acids and imidoyl chloride intermediates, which were generated through isocyanide-Nef reactions of acyl chlorides and (*N*-isocyanimine)triphenylphosphorane in CH_2_Cl_2_ at room temperature (Scheme 32).

**Scheme 33:**
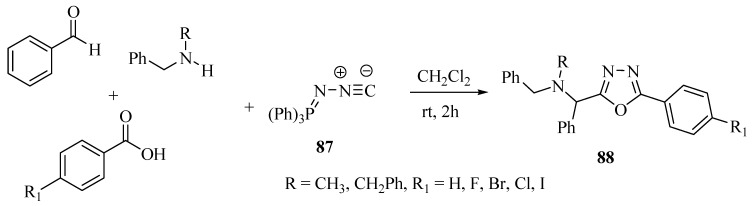
Synthesis of dissubstituted 1,3,4-oxadiazoles from four components in a one-pot procedure.

Ramazani and Rezaei [51] developed a novel and efficient method for the synthesis of 2,5-disubstituted-1,3,4-oxadiazoles **88** in high yield by a one-pot condensation procedure in CH_2_Cl_2_ at room temperature involving four components: (*N*-isocyanimino)triphenylphosphorane (**87**), a secondary amine, a carboxylic acid, and an aromatic aldehyde (Scheme 33). For similar reactions with their respective mechanisms, see references [52,53,54,55,56,57].

Although less popular than the methods mentioned above, the Huisgen reaction (reaction of 5-aryl/acyltetrazoles with acid chlorides or acid anhydrides) is widely used for the synthesis of various 2,5-disubstituted-1,3,4-oxadiazoles. Some interesting examples are outlined below.

**Scheme 34:**
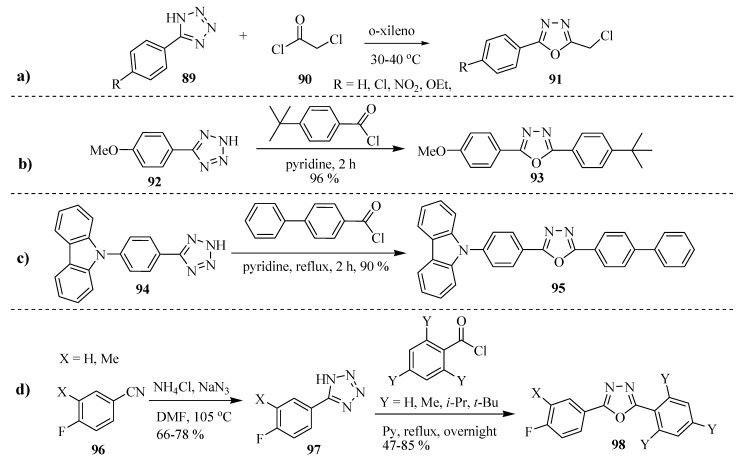
Synthesis of 1,3,4-oxadiazoles by the Huisgein reaction.

Tetrazole **89** with chloroacetyl chloride (**90**) give disubstituted oxadiazole **91**(Entry **a**, Scheme 34) [58]. Reflux of 4-methoxyphenyltetrazole (**92**) with 4-*tert*-butylbenzoyl chloride for 2 h affords 2-(4-*tert*-butylphenyl)-5-(4-methoxyphenyl)-1,3,4-oxadiazole (**93**) in 96% yield (Entry **b**, Scheme 34) [59]. Similarly, compounds **95** (Entry **c**) [60] and **98** (Entry **d**) [61] are obtained in excellent yields when treating the intermediates **94** and **97** with acid chlorides (Scheme 34). 

**Scheme 35:**
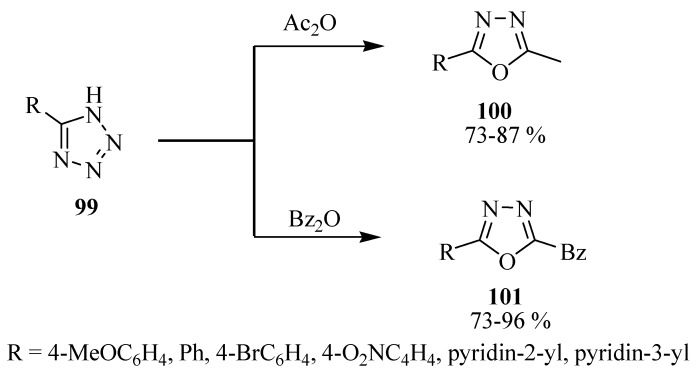
Acylation of tetrazoles with acetic and benzoic anhydrides.

The Huisgein reaction also proceeds well with acid anhydrides in place of acid chlorides, which was demonstrated by Efimova and co-workers [62], by synthesizing 1,3,4-oxadiazole compounds **100**, and **101** by acylation of a series of 5-aryl(hetaryl)tetrazoles **99** with acetic and benzoic anhydrides under microwave irradiation conditions (Scheme 35) (see also Reichart and Kappe [63]).

## 3. Pharmacological Activity of 1,3,4-Oxadiazoles

### 3.1. Antimicrobial Activity

The recent emergence of drug resistance when treating infectious diseases has underlined the need for new, safer, and more efficient antimicrobial agents. Many researchers have reported excellent antimicrobial activity for compounds containing the 1,3,4-oxadiazole core. 

**Figure 5:**
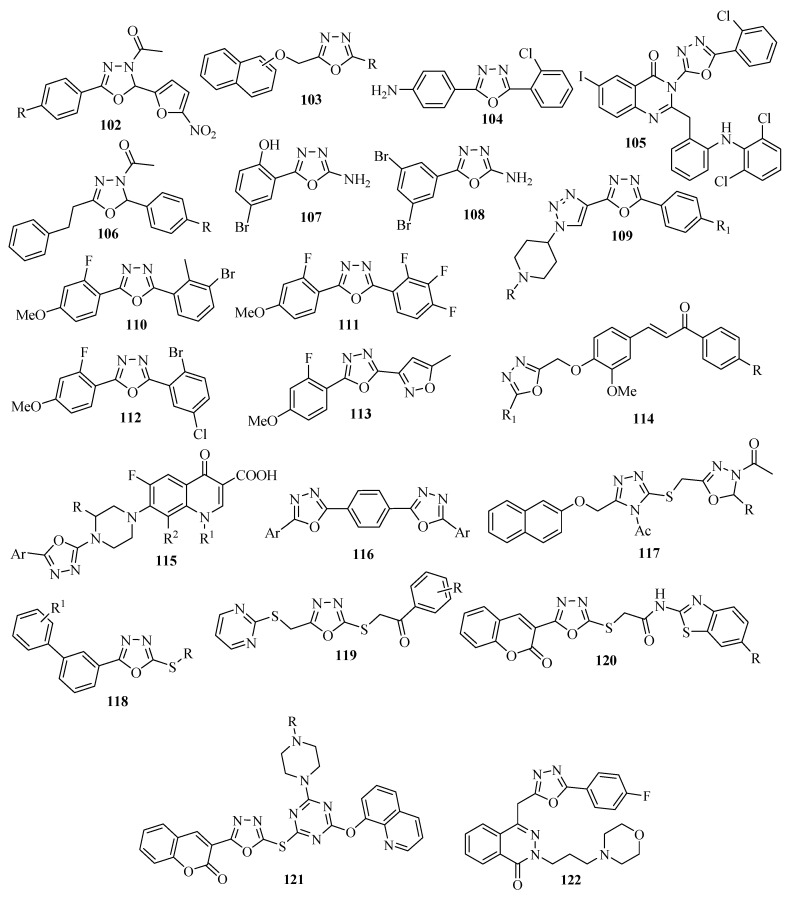
Disubstituted-1,3,4-oxadiazoles with antibacterial and antifungal activity.

Recently, Oliveira and co-workers [64] reported synthesis and antistaphylococcal activity of 1,3,4-oxadiazolines **102** against strains of *Staphylococcus aureus*, resistant to methicillin and amino glycosides (MARSA), and that encode efflux proteins (multidrug drugs resistant—MDR). The compounds **102** showed efficient antistaphylococcal activity at 4 to 32 μg/mL, making all the compounds 2–8 times more active than the standard drug chloramphenicol (Figure 5).

A series of new derivatives of 5-(1-/2-naphthyloxymethyl)-1,3,4-oxadiazol-2(3H)-thione (R=SH), 5-(1-/2-naphthyloxymethyl)-1,3,4-oxadiazole-2-amino (R=NH_2_), and 5-(1-/2-naphthyloxymethyl)-1,3,4-oxadiazol-2(3H)-ones (R=OH) **103** [65] were synthesized and evaluated for their antimicrobial activity. All were active against *S. aureus*, *E. coli*, *P. aeruginosa*, *C. albicans* and *C. parapsilosis* at a minimum concentration of 64–256 mg/mL (Figure 5).

Patel and Patel [6] verified the antibacterial activity of a series of derivatives containing the 1,3,4-oxadiazole nucleus against Gram-positive (*S. aureus* MTCC 96 and *S. pyogenes* MTCC 442) and Gram-negative bacteria (*E. coli* MTCC 443 and *P. aeruginosa* MTCC 1688) using ampicillin as the drug standard. The compounds 4-[5-(2-chlorophenyl)-1,3,4-oxadiazol-2-yl]benzenamine (**104**), and 3-{[5-(2-chlorophenyl)-1,3,4-oxadiazol-2-yl]methyl}-2-{2-[2,6-dichlorophenyl)amino]benzyl}-6-iodoquinazolin-4(3H)-one (**105**) were respectively 2 and 5 times more potent than ampicillin (Figure 5).

2,5-Disubstituted oxadiazole compounds **106** (Figure 5) containing the acetyl group in position 3 of the oxadiazole ring were synthesized and evaluated against two strains of bacteria, *S. aureus* and *P. aeruginosa*, and against two species of fungi, *C. albicans* and *A. flavus*, by the disk diffusion method. Ampicillin and fluconazole were used as drug standards for the antibacterial and antifungal activity, respectively. In comparison, all of the compounds were equally potent to their ampicillin and fluconazole standards [66].

The antibacterial and antifungal activity of 2-(5-amino-1,3,4-oxadiazol-2-yl)-4-bromophenol (**107**), and 5-(3,5-dibromophenyl)-1,3,4-oxadiazol-2-amine (**108**) were investigated against two strains of Gram-positive bacteria; *Streptococcus aureus*, *Bacillus subtilis*, two strains of *Gram-negative* bacterial; *Klebsiella pneumoniae* and *Escherichia coli*, and two fungal species; *Aspergillus Niger* and *C. Pannical.* The tests showed activities which were approximately equal to the standard drugs of treatment streptomycin and griseofulvin, respectively, [67] (Figure 5).

Sangshetti and co-workers [68] investigated the antifungal activity of a number of disubstituted oxadiazoles **109** (Figure 5), each of which contained a triazole unit at position 5 of the oxadiazole ring. The species of fungi tested were *Candida albicans*, *Fusarium oxysporum*, *Aspergillus flavus*, *Aspergillus Níger*, and *Cryptococcus neoformans*. Miconazole and fluconazole were used as standards for the comparison. The compounds containing the methyl sulfone (R=SO_2_CH_3_) group attached to the nitrogen of the piperidine ring, and Cl or OH (R_1_) groups exhibited excellent pharmacological profiles (equal to miconazole) against some of the fungi. 

Compounds **110** and **111** were respectively 2 and 4 fold more potent than furacin when evaluated against *E. coli*, and *P. aeruginosa*. Compounds **112** and **113** were twice as potent as fluconazole against *C. albicans* [69] (Figure 5). Other oxadiazole compounds with antibacterial activity are: **114** [70], **115** [71], **116** [72], **117** [13], **118** [73], **119** [24], **120** [74], **121** [75] and **122** [76] (Figure 5).

**Figure 6:**
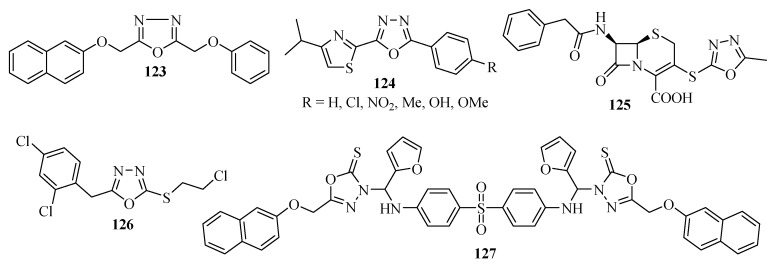
1,3,4-Oxadiazoles with anti-mycobacterial activity.

The compound 2-(2-naphthyloxymethyl)-5-phenoxymethyl-1,3,4-oxadiazole (**123**) exhibitsanti-mycobacterial activity at a minimum inhibitory concentration of 6.25 µg/mL (Figure 6) [77]. Anti-mycobacterial activity against *Mycobacterium tuberculosis* H_37_RV was also studied by Kumar and co-workers [40] for a series of di-substituted oxadiazoles **124** containing the thiazole unit. The derivative containing the Cl group exhibited excellent results at a minimum inhibitory concentration of 4 μg/mL (Figure 6).

Yoshida and co-workers [78] described the synthesis and optimization of anti-*Helicobacter pylori* activity for a new series of cephem derivatives. Compound **125** exhibited anti *Helicobacter pylori* (13001 and FP1757) activity at a minimum inhibitory concentration of 0.1 μg/mL. Bakal and Gattani [79] investigated anti-tubercular activity for a series of 2,5-disubstituted oxadiazoles against *M. tuberculosis* H_337_Rv. Compound **126** with a MIC_50_ = 0.04 ± 0.01 μM was comparable with Isoniazid. Compound **127** was 7.3-fold more active against *Mycobacterium tuberculosis* H_37_Rv, and 10.3-fold more active against INH resistant *Mycobacterium tuberculosis* than Isoniazid (Figure 6) [80].

### 3.2. Anticonvulsant Activity

**Figure 7:**
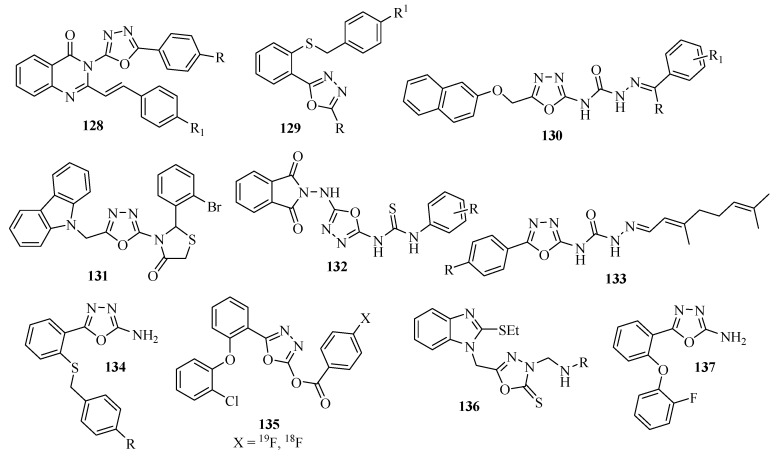
1,3,4**-**Oxadiazoles with anticonvulsant activity.

New 3-[5-(4-substituted)-phenyl-1,3,4-oxadiazole-2-yl]-2-styrylquinazoline-4(3H)-one oxadiazoles **128** were synthesized and evaluated by Kashaw and co-workers [81] (Figure 7) for anticonvulsant activity. New 2-substituted-5-(2-benzylthiophenyl)-1,3,4-oxadiazole derivatives **129** were designed and synthesized as anticonvulsant agents. The authors found that introduction of an amino group at position 2 of the 1,3,4-oxadiazole ring, and a fluorine substitute at the *para* position of the benzylthio group improves anticonvulsant activity [82], (Figure 7).

Rajak and co-workers [83] synthesized and evaluated semicarbazones **130** containing the 1,3,4-oxadiazole units for anticonvulsant potential in a three model test (MES), (scPTZ) and (scSTY). Most of them showed activity in all three models, (Figure 7). We include other compounds with anticonvulsant activity: **131** [84], **132** [85], **133** [86], **134** [82], **135** [87], **136** [88], **137** [89] (Figure 7).

### 3.3. Anti-inflammatory Activity

**Figure 8:**
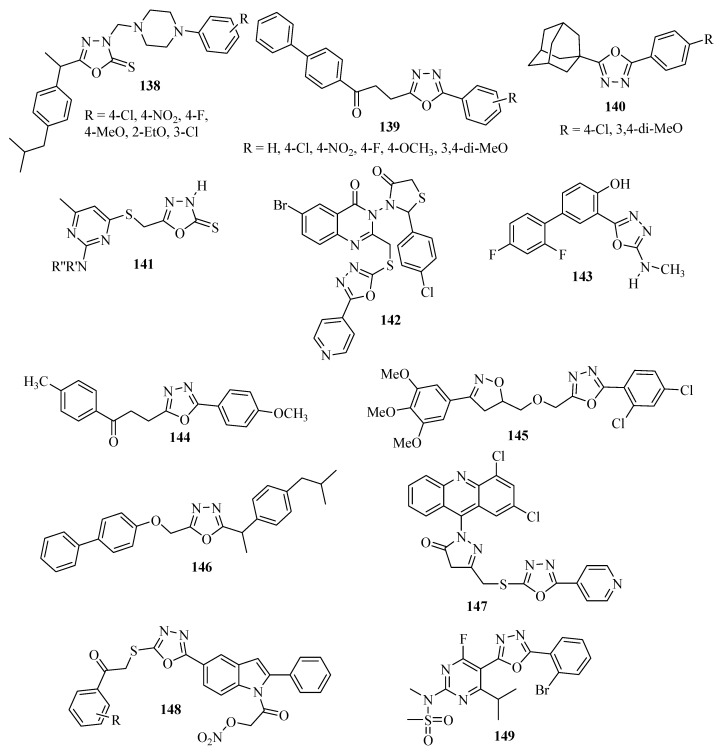
1,3,4-Oxadiazoles with anti-inflammatory activity.

A series of oxadiazole derivatives **138** of ibuprofen which contains the arylpiperazine unit at position 3 of the oxadiazole ring were investigated by Manjunatha and co-workers [20] for anti-inflammatory activity using paw edema induced by carrageenan as the method with sodium diclofenac as the reference. Compounds containing 4-Cl, 4-NO_2_, 4-F and 3-Cl groups were more active than sodium diclofenac, whereas compounds with 4-MeO and 2-EtO groups showed less activity (Figure 8).

Compounds **139** were synthesized from the anti-inflammatory drug fenbufen and evaluated for anti-inflammatory activity by carrageenan induced paw edema; sodium diclofenac and fenbufen were the standards. The compounds containing 4-Cl, 4-NO_2_, 4-F and 4-MeO groups were equipotent to fenbufen, and the compound with a 3,4-di-MeO group was more potent than the fenbufen, and equal to sodium diclofenac [90] (Figure 8).

2-(1-adamantyl)-5-substituted-1,3,4-oxadiazole compounds **140** displayed strong dose dependent inhibition of carrageenan-induced paw edema with >50% inhibition at a concentration of 60 mg/kg. The compound with the 3,4-di-MeO group was more potent than the indomethacin standard [91], (Figure 8). Burbuliene and co-workers [92] investigated anti-inflammatory activity for 5-[(2-di-substituted diamino-6-methyl-pyrimidin-4-yl)sulphanylmethyl]-3*H*-1,3,4-oxadiazol-2-thione derivatives **141** and found that some were more potent than ibuprofen, (Figure 8). Other compounds with anti-inflammatory activity are also included: **142** [93], **143** [94], **144** [95], **145** [96], **146** [97], **147** [98], **148** [99] and **149** [100], (Figure 8).

### 3.4. Analgesic Activity

**Figure 9:**
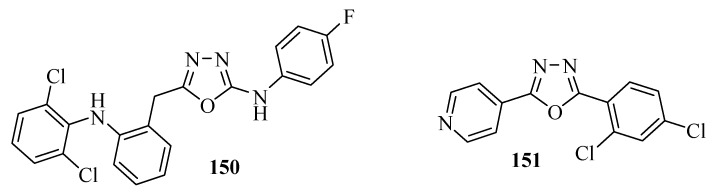
1,3,4-Oxadiazoles with analgesic activity.

5-(2-(2,6-Dichlorophenylamino)benzyl)-*N*-(4-fluorophenyl)-2-amino-1,3,4-oxadiazole (**150**) was more potent in an evaluation of its analgesic activity than sodium diclofenac with a maximal analgesic activity of (81,86%) [27] (Figure 9). The compound **151** containing the 2,4-dichlorophenyl group, present at the second position of the oxadiazole ring, showed a maximal activity of (70.37 ± 1.67%), almost equivalent to that of the ibuprofen standard (73.52 ± 1.00%) [101] (Figure 9). Compounds **138**, **139**, **144**, **146**, **147**, **148** (Figure 8) also display analgesic activity. Compound **139** with the R=4-F group showed a maximal analgesic activity of (72.52%), better than both sodium diclofenac (70.32%) and fenbufen (54.1%) [90].

### 3.5. Antitumor Activity

**Figure 10:**
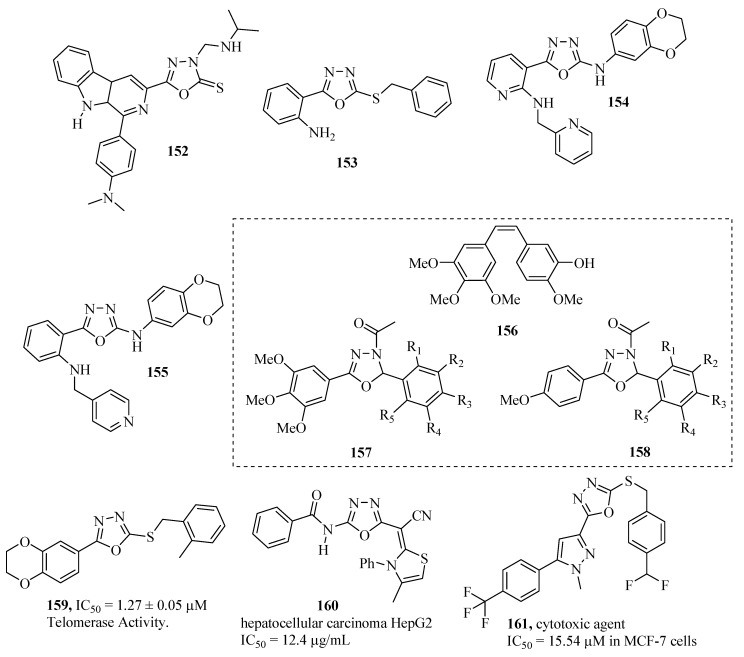
1,3,4-Oxadiazoles with antitumor activity.

Savariz and co-workers [102] synthesized and evaluated the *in vitro* antitumor activity of new Mannich bases. Among the compounds studied, compound **152** showed potent activity against melanoma (UACC-62), and lung (NCI-460) cell lines with GI_50_ values of 0.88 and 1.01 mmol/L, respectively, (Figure 10). Liu and co-workers [103] synthesized and reported the anti-proliferative and EGFR inhibition properties of a series of 2-(benzylthio)-5-aryloxadiazole derivatives. Compound **153** showed potent biological activity (IC_50_ = 1.09 μM for MCF-7, and IC_50_ = 1.51 μM for EGFR) (Figure 10).

Ouyang and co-workers [104], and Tuma and co-workers [105] synthesized and evaluated various 1,3,4-oxadiazole derivatives as to their ability to inhibit tubulin polymerization and block the mitotic division of tumor cells. Compounds **154** and **155** exhibited potent activity. *In vitro* studies of compound **154** indicated that at nano-concentrations it interrupted mitotic division in breast carcinoma and squamous cell tumors, which included multi-drug resistant cells. *In vivo* studies of compound **155** displayed a desirable pharmacokinetic profile (with appropriate plasma levels after oral administration), and was significantly more effective than the taxane paclitaxel (Figure 10).

The anti-proliferative effects of 24 new 2,5-diaryl-2,3-dihydro-1,3,4-oxadiazoline compounds (Type I) **157**, and (Type II) **158**, analogous to combretastatin-A4 (**156**) were evaluated in murine L1210 leukemia cells (Figure 10), and also in murine B16 melanoma cells. Combretastatin-A4 is the most potent of natural combretastatins. Early studies have shown that it inhibits tubulin polymerization, and proliferation of murine, and human cancer cells. Type I compounds with R_1_=R_2_=R_4_=R_5_=H, and R_3_=Br groups, displayed an IC_50_ of 0.6 ± 0.7 μM. Type II compounds with R_1_=R_5_=H, and R_2_=R_3_=R_4_=OCH_3_ displayed an IC_50_ of 0.5 ± 0.06 μM. However, these compounds were substantially less potent than the compound **156** which displayed an IC_50_ of 0.003 μM (all for L1210 cells) [106]. Other compounds with antitumor activity are included: **159** [107], **160** [108] and **161** [109].

### 3.6. Antiviral Activity

**Figure 11:**
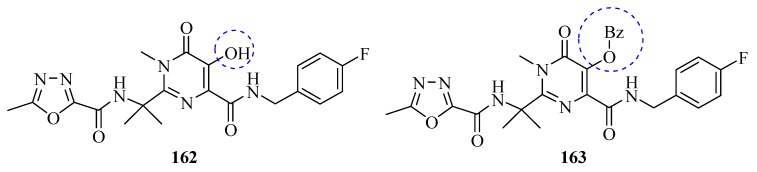
Structures of raltegravir (**162**) and derivatives.

On October 16, 2007, the US Food and Drug Administration (FDA) approved raltegravir (Isentress^®^, **162**, Figure 11) for treatment of human immunodeficiency virus (HIV)-1 infection, in combination with other antiretroviral agents in treatment-experienced adult patients who have evidence of viral replication, and HIV-1 strains resistant to multiple antiretroviral agents. Raltegravir is the prototype of a new class of antiretroviral drugs known as integrase inhibitors [110].

Seeking to identify more promising compounds than raltegravir, Wang and co-workers [111] synthesized a series of raltegravir derivatives by modifying the 5-hydroxyl group of the pyrimidine ring and evaluated them for anti-HIV activity. The 5-hydroxyl modification of raltegravir derivatives significantly increased their activity, which indicates the 5-hydroxyl group’s dispensability. Compound **163** with a sub-picomol IC_50_ value was the most potent anti-HIV agent among all of the derivatives synthesized, and thus emerged as a new and potent anti-HIV agent (Figure 11).

**Figure 12:**
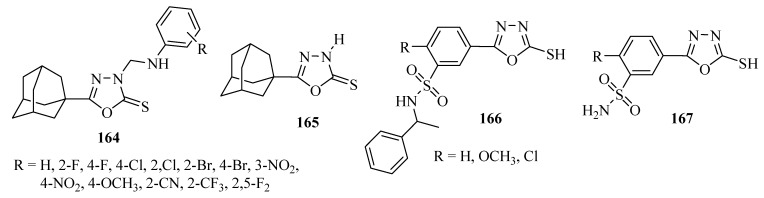
1,3,4-Oxadiazoles with inhibitory activity against human immunodeficiency virus type 1 (HIV-1).

The inhibitory activity of the compounds **164** and **165** (Figure 12) against the human immunodeficiency virus type 1 (HIV-1) was determined using the XTT assay on MT-4 cells. Compound **165** was the most active among the compounds tested, producing 100, 43 and 37% reductions in viral replication at concentrations of 50, 10 and 2 µg/mL respectively. Compounds **164** with R=4-F, and 2-Br groups exhibited less anti-viral replication activity yet above 10% inhibition at concentrations of 2 µg/mL. All tested compounds were non-cytotoxic with CD_50_ > 100 µg/mL except compound **165** whose CD_50_ was 68 µg/mL [112].

Iqbal and co-workers [113] reported inhibitory activity for compounds **166** and **167** (Figure 12) against the human immunodeficiency virus type 1 (HIV-1) which was also determined using the XTT assay on MT-4 cells. Compound **166** with the R=Cl group was the most active among the compounds tested, with 62, 21 and 14% reductions at concentrations of 50, 25 and 5 μg/mL, respectively.

**Figure 13:**
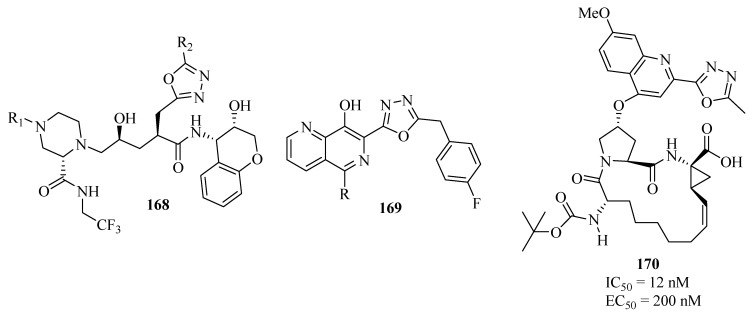
1,3,4-Oxadiazoles with inhibitory activity against HIV and hepatitis C virus.

Indinavir, another protease inhibitor is also used as a component of antiretroviral therapy for treating HIV infection and AIDS. Kim and co-workers [114] have synthesized and evaluated the protease inhibitory activity of a series of oxadiazoles **168** analogous to indinavir. All the compounds prepared inhibited protease activity at picomolar (IC_50_) concentrations (thus being more potent than the indinavir) (Figure 13).

Johns and co-workers [115] reported antiviral activity (through inhibition of viral DNA integration) for new derivatives containing the 1,3,4-oxadiazole unit in combination with a ring system of 8-hydroxy-1,6-naphthyridine **169**. Compound **170**, containing a 5-methyl-1,3,4-oxadiazol-2-yl group at the C2 position of the quinoline ring, shows inhibitory activity against the hepatitis C virus NS3 protease [116] (Figure 13).

### 3.7. Antihypertensive Activity

**Figure 14:**
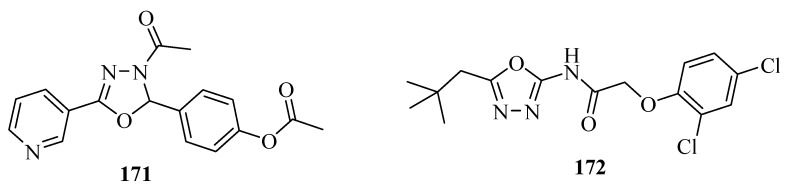
Vasorelaxant activity of 1,3,4-oxadiazoles.

Hypertension and cardiovascular disease are major causes of morbidity and mortality worldwide. Bankar and co-workers [117] reported the vasorelaxant effect of compound **171, ** 4-(3-acetyl-5-(pyridin-3-yl)-2,3-dihydro-1,3,4-oxadiazol-2-yl)phenyl acetate (Figure 14), in rat aortic rings by blocking L-type calcium channels. Bankar and co-workers [118] also investigated whether the correction of endothelial dysfunction is dependent on high blood pressure normalization; in deoxycorticosterone acetate (DOCA-salt), and N^G^-nitro-L-arginine (L-NNA) in hypertensive rats. Compound **172** is a T type Ca^2+^ channel inhibitor with an IC_50_ of 810 nM [119] (Figure 13).

### 3.8. Enzyme Inhibitors

**Figure 15:**
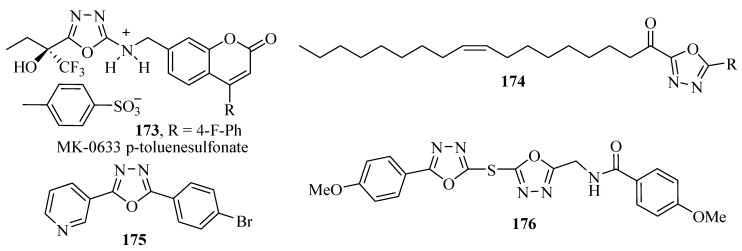
1,3,4-Oxadiazole as enzyme inhibitors.

Leukotrienes (LTs) are potent inflammatory lipid mediators derived from arachidonic acid metabolism, and released from cells involved in inflammation. The synthesis of all LTs requires the action of the enzyme 5-lipoxygenase (5-LO). Inhibition of 5-LO reduces the production of both LTB_4,_ and cysteinyl LTs (CysLTs); LTC_4_, LTD_4_ and LTE_4_. 5-LO inhibitors have therapeutic potential for the treatment of inflammatory processes. A new oxadiazole p-toluenesulfonate derivative **173** (Figure 15) containing an asymmetric carbon was identified as both a potent and selective inhibitor of 5-lipoxygenase (5-LO) by Ducharme and co-workers [120], and Gosselin and co-workers [121].

Leung and co-workers [122] reported the discovery of a new class of disubstituted oxadiazoles **174** (Figure 15) from oleic acid derivatives with potent and selective inhibition of fatty acid amide hydrolase. Khan and co-workers [123] performed studies on inhibition effects on tyrosinase with 19 2,5-disubstituted-1,3,4-oxadiazole compounds, the compound 3-(5-(4-bromophenyl)-1,3,4-oxadiazol-2-yl) pyridine (**175**) with an IC_50_ of 2.18 µM was more potent than the standard L-mimosine (IC_50_ = 3.68 µM) (Figure 15).

Tomi and co-workers [124] reported a study with the bis-1,3,4-oxadiazole compound **176** that contains a glycine unit on the transferase activity of enzymes such as: GOT, GPT and γ-GT in serum. Compound **176** showed activation for GOT and GPT and inhibitory effects on the activity of γ-GT, (Figure 15).

**Figure 16:**
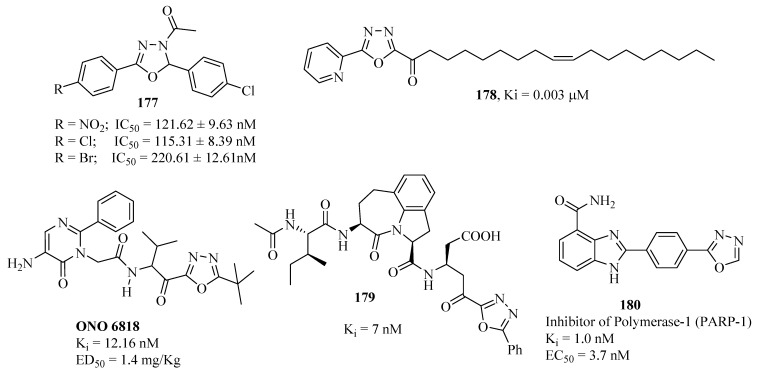
1,3,4-Oxadiazoles showing inhibitors activity against enzymes.

Maccioni and co-workers [125] synthesized a set of 3-acetyl-2,5-diaryl-2,3-dihydro-1,3,4-oxadiazoles and tested them as inhibitors of human monoamine oxidase (MAO) A and B isoforms. None of the tested compounds displayed significant inhibitory ability for MAO-A. However, several compounds were identified as selective MAO-B inhibitors. Some of the tested compounds exhibit interesting biological properties with an IC_50_ for the B isoform ranging from micromolar to nanomolar values. Compounds **177** were active at inhibiting MAO-B at nanomolar concentrations (Figure 16).

The optimization study of the central heterocycle of α-ketoheterocycle inhibitors of fatty acid amide hydrolase realized by Garfunkle and co-workers [126], led to identification of the most potent inhibitors **178**. 5-aminopyrimidinone R-keto-1,3,4-oxadiazole (**ONO-6818**) is representative of orally active nonpeptidic reversible inhibitors of Human Neutrophil Elastase (HNE), with potent Ki values in the nanomolar range (Figure 16) [127,128]. Selective human Granzyme B inhibitors **179** inhibit CTL mediated apoptosis [129], and compound **180** (EC_50_ = 3.7 nM) demonstrated balanced potency and PK profiles. The molecule also exhibited potent oral *in vivo* efficacy potentiating the cytotoxic agent temozolomide in a B16F10 murine melanoma model [130], (Figure 16).

**Figure 17:**
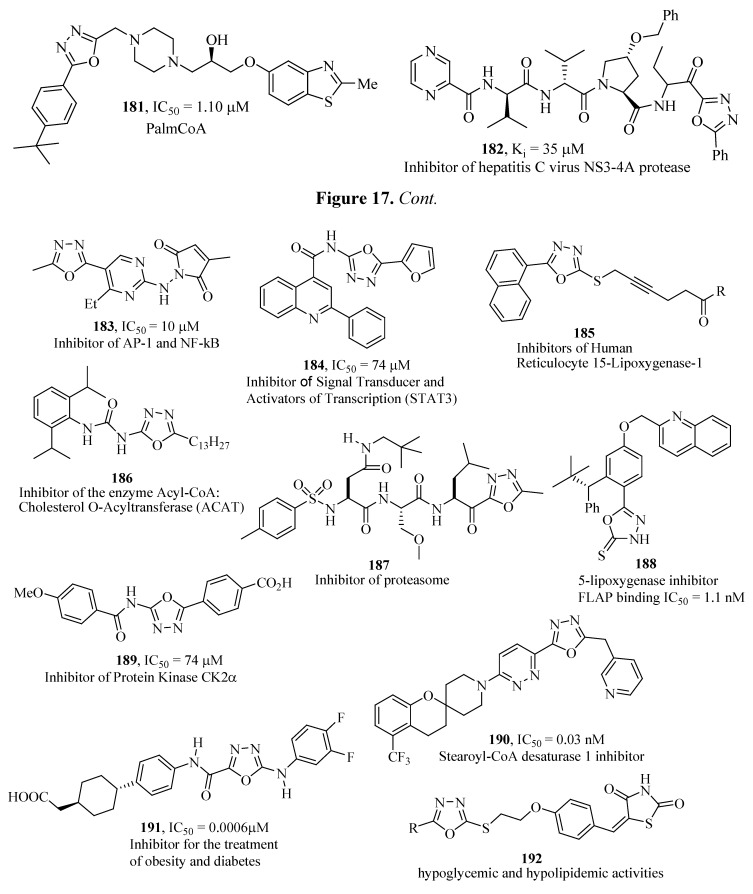
Other 1,3,4-oxadiazole derivatives as enzymes inhibitors.

Various other 1,3,4-oxadiazole derivatives show inhibitory activity against enzymes such as: **181** [131], **182** [132], **183** [133], **184** [134], **185** [135], **186** [136], **187** [137], **188** [138], **189** [139], **190** [140], **191** [141] and **192** [142], (Figure 17).

**Figure 18:**
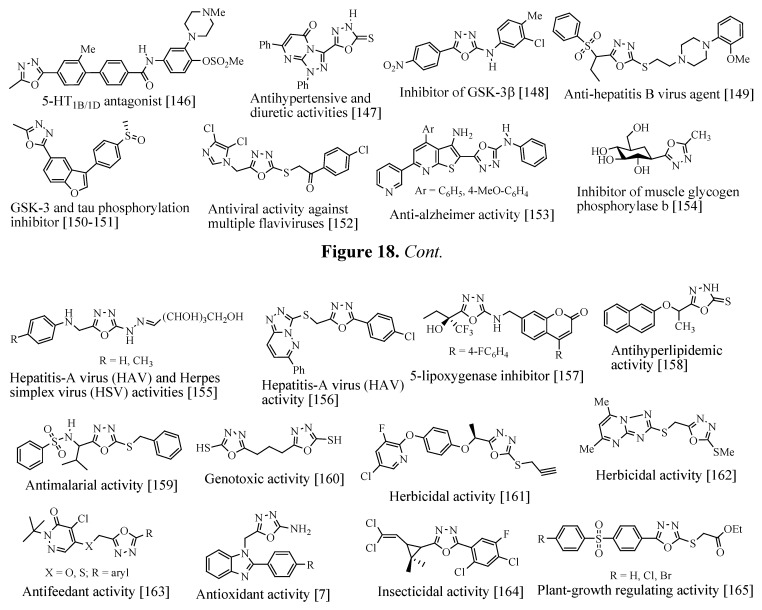
Other biological and pharmacological activities of 1,3,4-oxadiazoles derivatives.

A summary of other activities exhibited by compounds containing the 1,3,4-oxadiazole nucleus can be found in the Figure 18. For further biological and pharmacological properties of 1,3,4-oxadiazole, see references [143,144,145].

## 4. Conclusions

This review, has summarized the synthetic methods and biological activities for 1,3,4-oxadiazole derivatives reported in the literature during the past twelve years. The main synthetic methods include: (1) cyclodehydration reactions of diacylhydrazines; (2) cyclization oxidative reactions of *N*-acylhydrazones; (3) the one-step synthesis from readily available carboxylic acids and acid hydrazides; (4) the reactions of hydrazides with orthoesters; (5) hydrazide reactions with carbon disulfide in basic medium; (6) reaction of tetrazoles with acid chloride or acid anhydride. Most research groups are still using these synthetic routes making use only of new reaction conditions such as: new cyclization reagents, new catalysts, polymeric supports and microwave radiation. Few innovative methods have emerged in recent years, highlighting the methods described by Ramazani and Rezaei [51] and Cui and co-workers [50]. Furthermore, the various synthetic methods exemplified may serve as a support for the planning of new molecules containing the 1,3,4-oxadiazole unit. The broad pharmacological profile of this class of compounds is evidenced by the numerous examples cited here. In each biological activity topic, we have only provided selected examples of molecules with relevant activity being that these molecules may serve as prototypes for the development of more active derivatives.
